# Food provisioning to *Pardosa* spiders decreases the levels of tissue-resident endosymbiotic bacteria

**DOI:** 10.1038/s41598-023-34229-1

**Published:** 2023-04-28

**Authors:** Milan Řezáč, Veronika Řezáčová, Nela Gloríková, Ema Némethová, Petr Heneberg

**Affiliations:** 1 Crop Research Institute, Drnovská 507, 160 00 Prague, Czech Republic; 2 Charles University, Third Faculty of Medicine, Ruská 87, 100 00 Prague, Czech Republic

**Keywords:** Entomology, Microbiome

## Abstract

The diversity, host specificity, and physiological effects of endosymbiotic bacteria in spiders (Araneae) are poorly characterized. We used 16S rDNA sequencing to evaluate endosymbionts in the cephalothorax and legs of a wolf spider *Pardosa agrestis*. We tested the effects of feeding once or twice daily with fruit flies, aphids, or starved and compared them to those of syntopically occurring *Pardosa palustris*. The feeding increased traveled distance up to five times in some of the groups provisioned with food relative to the starved control. The Shannon diversity *t*-test revealed significant differences between these component communities of the two spider species. The increased frequency of feeding with fruit flies, but not aphids, increased the dominance and decreased the alpha diversity of OTUs. The obligate or facultative endosymbionts were present in all analyzed spider individuals and were represented mostly by *Rickettsiella*, *Rhabdochlamydia*, *Spiroplasma*, and the facultative intracellular parasite *Legionella*. Vertically transmitted endosymbionts were less common, represented by *Wolbachia pipientis* and *Rickettsia* sp. H820. The relative abundance of *Mycoplasma* spp. was negatively correlated with provisioned or killed aphids. In conclusion, the tissues of *Pardosa* spiders host tremendously diverse assemblages of bacteria, including obligate or facultative endosymbionts, with yet unknown phenotypic effects.

## Introduction

Spiders are important predators that are capable of repopulating disturbed ecosystems, including crop fields. In European crop fields, spiders have high density^1^. The central European arable fields, particularly their margins, are dominated by Lycosidae^2,3^. The prey of Lycosidae in crop fields consists prevalently of dipterans, aphids, and springtails^4^. The removal of spiders leads to an increase in these insects, including economically important aphids^5–7^. Intensively disturbed ecosystems, such as crop fields, are characterized by outbreaks of specific prey types^8,9^. Nevertheless, the effects of exposure to different prey types and to different prey quantities are not well understood beyond simple satiety, fecundity, and survival experiments^10–12^. Whether differences in prey type and quantity affect tissue-resident spider microbiome is unclear. Data from other invertebrates suggest that tissue-resident endosymbiotic organisms respond swiftly to diet-induced changes^13–16^. However, altered quantity of endosymbiotic organisms, like *Wolbachia*, has often only limited or no effect on the nutrition, fecundity or viability of their invertebrate hosts^13,17^. The sublethal effects may be difficult to identify and axenic models of invertebrates (such as^18–21^) are needed, allowing studies of similar nature as performed in vertebrates (e.g.,^22–24^).

The structure and dynamics of spider microbiomes remain enigmatic. Several studies are available regarding the gut microbiome (e.g.,^25–30^). Some other studies examined the microbiomes of whole spider bodies without reflecting the tissue specificity of the microbes (e.g.,^31,32^) or investigated the microbiome of the whole abdomen^33^. The latter study concluded that there is a difference between starved and fed spiders of multiple species^33^. The microbial endosymbionts were recently studied using 16S rDNA sequencing in the invasive *Argiope bruennichi* spider^34^, which identified differences between populations and individuals but not between examined tissue types—the study examined legs, prosoma, hemolymph, book lungs, ovaries, silk glands, midgut, and fecal pellets. Similarly, a study on five Theridiidae spp. suggested phylosymbiosis of the endosymbionts across the examined tissues and suggested congruence with host phylogeny^35^. Previous studies reported abundant endosymbionts across multiple spider families, documenting the presence of reproductive parasites, such as *Wolbachia*, *Cardinium*, *Rickettsia*, *Spiroplasma*, and *Rickettsiella*^36–39^.

The extent of the phenotypic effects induced by microbial endosymbionts in spiders is unknown. However, some exceptions apply, and *Wolbachia* has been associated with a skewed sex ratio in *Oedothorax gibbosus*^40^, *Rickettsia* has been associated with changes in the dispersal of *Erigone atra*^41^, *Ricketsiella* has been shown to induce cytoplasmic incompatibility in *Mermessus fradeorum*^42^, and *Rhabdochlamydia* has been shown to have different infection intensities in females and males in *O. gibbosus*^39^. It remains to be analyzed whether the food composition and food quantity have any effects on the species richness and relative abundance of endosymbionts and whether there exists a feedback loop that leads to higher predation rates or higher locomotor activity. Locomotor activity is known to be affected in other invertebrates, in which *Wolbachia* commonly affects host activity, but the direction of these effects is unpredictably variable and sometimes dependent on host sex^43–45^; the data from spiders are lacking. Environmental factors have already been shown to correlate with differences in microbiome composition in *Stegodyphus dumicola*^46^. The only study that addressed the effects of microbiota on foraging behavior was performed using the same spider species, which has been exposed to increased loads of cuticular bacteria *Bacillus thuringiensis*, *Microbacterium oxydans*, and *Pantoea* sp. The study concluded that control individuals attacked prey more quickly and were more effective in their cooperative behavior relative to the exposed individuals^47^.

In the present study, we hypothesized that the species richness and relative abundance of microbial endosymbionts of lycosid spiders both affect and can be affected by feeding and that differences in colonization by microbial endosymbionts can project to changes in locomotor parameters. We therefore provisioned *Pardosa agrestis* with three types of diets (*Drosophila* flies, *Metopolopium* aphids, or starving), recorded the amount of killed prey, analyzed the microbiome of their legs and cephalothorax (thus avoiding the contamination with gut microbiota), analyzed differences in locomotor behavior, and compared the microbial colonization of the study species with syntopically occurring *P. palustris*.

## Materials and methods

### Experimental design

For the purpose of the present study, we collected and examined 25 adult females of *P. agrestis*. To test for the similarity of tissue-resident microbiomes between morphologically close syntopic species living at the same sampling site, we also collected and examined five adult females of *P. palustris*. All originated from certified organic wheat fields in Prague-Ruzyně, Czech Republic (50.08° N, 14.30° E). We chose to examine a single sex of spiders to reduce the variability among replicates. We placed the females individually into 60-mm Petri dishes with carbon plaster on the bottoms. Following the collection of females, they were acclimated for two weeks at 22 °C, 80% humidity, and a natural light/dark cycle; the experiment itself was performed under the same conditions. During acclimation, the spiders were not fed to ensure that all were equally starved at the beginning of the experiment. During the experiment, the females received 10 females of flightless mutants of *Drosophila melanogaster* or 30 *Metopolopium dirhodum* aphids once or twice daily or were not fed (control). While *D. melanogaster* is a characteristic laboratory prey provisioned to wolf spiders, *M. dirhodum* is a representative of characteristic natural prey of wolf spiders. The food was provisioned at 7:00 and 19:00 (for those fed twice daily). To enable the manifestation of changes in the microbiome, the treatment lasted seven days. Combined, we compared the effects of increased feeding frequency, the effects of different food types, and differences in the microbiome of the two closely related spider species. We assigned the tested spiders to individual treatment groups as follows:

1. *P. agrestis*, fed with aphids once daily—six individuals
2. *P. agrestis*, fed with aphids twice daily—four individuals
3. *P. agrestis*, fed with fruit flies once daily—five individuals
4. *P. agrestis*, fed with fruit flies twice daily—five individuals
5. *P. agrestis*, starved (control)—five individuals
6. *P. palustris*, fed with aphids twice daily (three individuals) or fruit flies once daily (two individuals), analyzed together—five individuals

We recorded the number of killed prey, and we tested differences in locomotor parameters. To test locomotion, we plugged 60-mm Petri dishes with translucent plugs to avoid the escape of spiders. We videotaped the spiders for 10 min with a Panasonic WV-CP480 SDIII-Super Dynamic camera. We used EthoVision tracking software to measure the behavior of spiders during the video-recorded trials^48^. For the purpose of the present study, we analyzed the total distance moved [cm].

### DNA extraction

We extracted DNA from the legs and cephalothorax of the tested spiders. In total, we analyzed 30 spider individuals representing two species and five treatment conditions. We fixed the whole spiders in 96% ethanol. We washed them for 1 min with 1 mL of 0.5% bleach and for another minute with 1 mL of sterile distilled water; the wash steps were repeated two times. We then tore the analyzed body parts of spiders with sterile tweezers or cut them with a scalpel. We extracted the DNA using the DNeasy Blood & Tissue Kit (Qiagen, Hilden, Germany) according to the manufacturer`s instructions.

### DNA amplification and sequencing

To identify the composition of bacterial and archaeal assemblages, we amplified the V4 variable region of 16S rDNA. We used a combination of ten forward 515F and nine reverse 806R primers^49,50^, which were tagged with 5–7 nt-long specific barcodes for multiplexing. The list of primers is provided in Table 1. These primers were recommended by the Earth Microbiome Project and have frequently been used to study microbiome assemblages^51,52^. We amplified the analyzed samples using equimolar mixtures of the primers and the TP HS DNA-free 2 × Master Mix (Top-Bio, Vestec, Czech Republic) with the following set-up: initial denaturation: 94 °C for 4 min, 35 cycles of 94 °C for 45 s, 50 °C for 40 s, and 72 °C for 75 s; and final elongation at 72 °C for 10 min. We checked the quantity of the resulting PCR products using Quantifluor ONE dsDNA System (Promega, Madison, WI) and purified the PCR products using the QIAquick PCR Purification Kit (Qiagen). We performed the PCR in triplicate for each sample and pooled the resulting purified amplicons prior to sequencing. We then sequenced equimolar amplicon libraries on a 2 × 300-bp MiSeq platform (Illumina, San Diego, CA). We used PCR water as a negative control for the presence of contaminants in the PCR.

**Table 1:** List of primers used in the present study.

	Primer_tag	Primer sequence
FW primers	515F_T001	AAAGCGTGTGYCAGCMGCCGCGGTAA
	515F_T002	ACGAAGTGTGYCAGCMGCCGCGGTAA
	515F_T003	ACCTTGTGTGYCAGCMGCCGCGGTAA
	515F_T005	ATAATGTGTGYCAGCMGCCGCGGTAA
	515F_T048	AATGCAGTGTGYCAGCMGCCGCGGTAA
	515F_T049	AATTTAGTGTGYCAGCMGCCGCGGTAA
	515F_T051	AGATTGGTGTGYCAGCMGCCGCGGTAA
	515F_T096	ATTGCGTGTGTGYCAGCMGCCGCGGTAA
	515F_T099	ATAAAGAGTGTGYCAGCMGCCGCGGTAA
	515F_T100	ATGCTGAGTGTGYCAGCMGCCGCGGTAA
RV primers	806R_T003	ACCTTCCGGACTACNVGGGTWTCTAAT
	806R_T007	AGCCACCGGACTACNVGGGTWTCTAAT
	806R_T011	AACAGCCGGACTACNVGGGTWTCTAAT
	806R_T014	ACGGCCCGGACTACNVGGGTWTCTAAT
	806R_T052	ATCCTCCCGGACTACNVGGGTWTCTAAT
	806R_T054	AACCCGCCGGACTACNVGGGTWTCTAAT
	806R_T101	ACGGCTCCCGGACTACNVGGGTWTCTAAT
	806R_T105	ACGACATCCGGACTACNVGGGTWTCTAAT
	806R_T107	ATCCCGCCCGGACTACNVGGGTWTCTAAT

### Data analyses

We processed the sequence reads using SEED 2.1.2^53^. We assembled the paired-end reads and removed the low-quality reads, short reads (< 295 bp), and long reads (> 315 bp). We then clustered the 16S rDNA sequences into OTUs by applying a 97% similarity threshold. We also checked for chimeras using the built-in USEARCH 8.1.1861 algorithm. To classify the identified OTUs into species and higher taxonomic units, we used the database based on 16S RDP Release 11, updated on March 28, 2022^54^. We pooled the reads affiliated with the same OTU. To analyze changes at the higher taxonomic levels, we pooled the OTUs according to the NCBI Taxonomy Browser^55^. For unknown genera, we indicated the name of the identifiable higher taxon, which could result in a slight underestimation of the total number of genera but affects all processed samples equally. We excluded the sequences of eukaryotic DNA from further analyses. The lowest number of reads per sample was 794; all samples were retained in the dataset because the lower numbers of sequences did not affect the calculation of relative ratios when comparing the treatments and host species.

In total, we identified 871,064 sequences (reads), from which we excluded 10,112 sequences that represented chimeric clusters and were removed from further analyses. We grouped the obtained sequences into 3697 clusters. We further decreased this number to 3606 clusters after removing eukaryotic sequences and non-16S rDNA sequences.

The average partitioning of the clusters among Archaea/Bacteria, no hits, and Eukaryota was 99.48%, 0.26%, and 0.26%, respectively. Note that these clusters were identified based on 97% sequence identity. Any currently known bacterial species could therefore be represented by multiple related clusters.

We subsequently analyzed the diversity of the detected OTUs, genera, and higher taxa using a panel of biodiversity indices. We calculated Gini-Simpson index, where 1 indicates complete domination of a single taxon and 0 indicates equal representation of all taxa. Fisher’s alpha is a parametric diversity measure assuming the abundance of a particular taxon follows the log series distribution. The Berger-Parker index is calculated as the number of individuals in the dominant taxon relative to the total number of individuals^56^. We employed one-way PERMANOVA to identify the differences among the tested treatments. We further used the nonmetric multidimensional scaling (NMDS) to analyze the effects of explanatory variables (species, prey type, feeding frequency, number of killed prey, and total distance moved). We determined the goodness of fit of the proposed NMDS solutions by quantifying the interpoint distances in comparison to the original dissimilarities using Shepard plots. We used the Spearman correlation coefficient to analyze the associations between the explanatory variables and individual taxa. We applied Bonferroni correction (at n = 6) to reduce the chances of obtaining type I errors. We used a one-tailed *t*-test to compare the diversity indices, traveled distance, and amount of killed prey; the comparisons were made between treatment groups of *P. agrestis*. We used the Shannon diversity *t*-test to compare the assemblages that were associated with *P. agrestis* and *P. palustris*. To estimate the completeness of analyzed datasets, we employed sample rarefaction of OTUs from *P. agrestis* and *P. palustris*, and individual rarefaction of phyla from these two host species. We performed all calculations in SigmaPlot 12.0 and PAST 2.14. Data are shown as the mean ± SEM unless stated otherwise.

## Results

### Prey killing and locomotion

**Figure 1:**
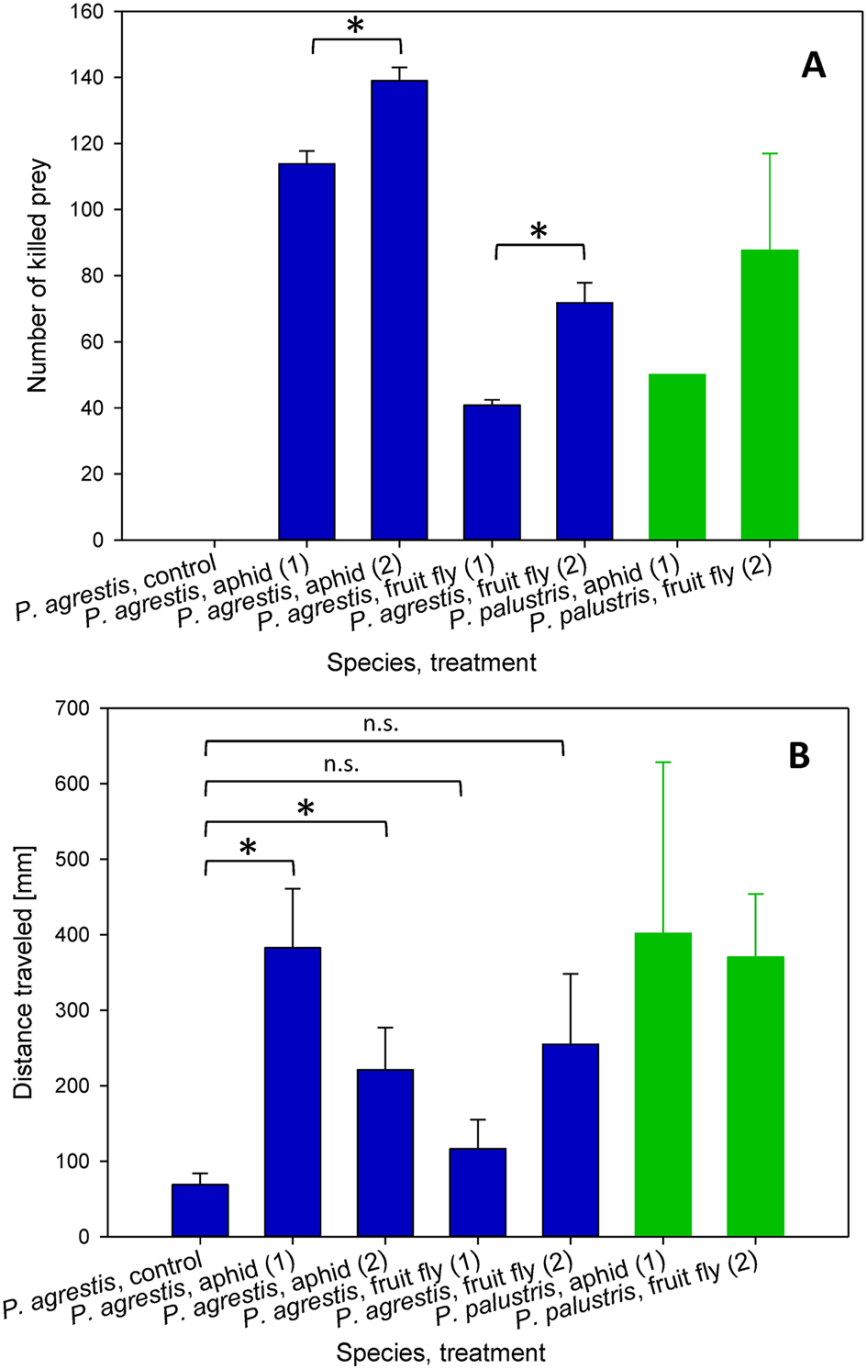
Number of killed prey (**A**) and traveled distance (**B**) in *P. agrestis* and *P. palustris* that were starved or fed at the indicated frequency with aphids or fruit flies. Asterisks indicate differences that were significant when tested using a one-sided *t*-test (*p* < 0.05), and “n.s.” indicates comparisons that were not significant (*p* ≥ 0.05).

*Pardosa agrestis* spiders consumed a higher total amount of prey when provisioned twice daily instead of once daily. This observation applied both to the number of killed aphids (113.8 ± 3.9 aphids vs. 139.0 ± 4.0 aphids during seven days when provisioned once or twice daily, respectively) as well as fruit flies (40.8 ± 1.6 fruit flies vs. 71.8 ± 6.1 fruit flies during seven days when provisioned once or twice daily, respectively) (Fig. 1A). Both differences were statistically significant (one-sided *t*-test *p* = 0.002 and *p* = 0.001, respectively).

*Pardosa agrestis* spiders that were provisioned with different amounts of food differed in total distance traveled. The control spiders, which were not provisioned with any food, traveled the shortest distance during the 10-min observation (69.0 ± 14.8 cm). In contrast, *P. agrestis* provisioned with aphids once daily traveled 382.6 ± 78.2 cm (one-sided *t*-test vs. control *p* = 0.005), and those fed two times daily also increased the traveled distance, but only to 221.1 ± 56.0 cm (one-sided *t*-test vs. control *p* = 0.02). The traveled distance also displayed increasing trends in *P. agrestis* fed fruit flies once daily (116.5 ± 38.8 cm) or twice daily (255.1 ± 93.2 cm) (Fig. 1B), but the differences were not significant (one-sided *t*-test vs. control *p* > 0.05 both).

### Bacterial and archaeal assemblages of *Pardosa* spp.

The analyzed assemblages consisted of 3601 OTUs of bacterial origin and five OTUs of archaeal origin. Of these OTUs, we retrieved 2801 bacterial OTUs and all five archaeal OTUs from *P. agrestis*, and 1190 bacterial OTUs and a single archaeal OTU from *P. palustris*. Rarefaction revealed that the analyzed assemblages of the two species were nearly complete with respect to higher taxa, e.g., phyla, but incomplete with respect to the amount of detected OTUs (Fig. S1). The Shannon diversity *t*-test revealed significant differences between these two assemblages (*t* = 120.08, *d*_*f*_ = 4.6487 × 10^5^, *p* < 0.001). The assemblage associated with *P. agrestis* had lower Gini-Simpson index (0.08 vs. 0.12) and higher Fisher’s alpha diversity (379.6 vs. 164.1). The number of OTUs reached 240.7 ± 14.1 (range 61–416) OTUs per host individual. The diversity of OTUs differed strongly when comparing *P. agrestis* fed fruit flies once and twice daily—the Gini-Simpson index was higher in spiders fed once daily (0.4 ± 0.1 vs. 0.1 ± 0.0), whereas the Simpson index, Shannon H, Brillouin, Menhinick, and Fisher’s alpha were higher in spiders fed twice daily (one-tailed *t*-test *p* < 0.05 each). In contrast, we did not detect such differences between *P. agrestis* fed aphids once or twice daily (one-tailed *t*-test *p* > 0.05 each).

**Figure 2:**
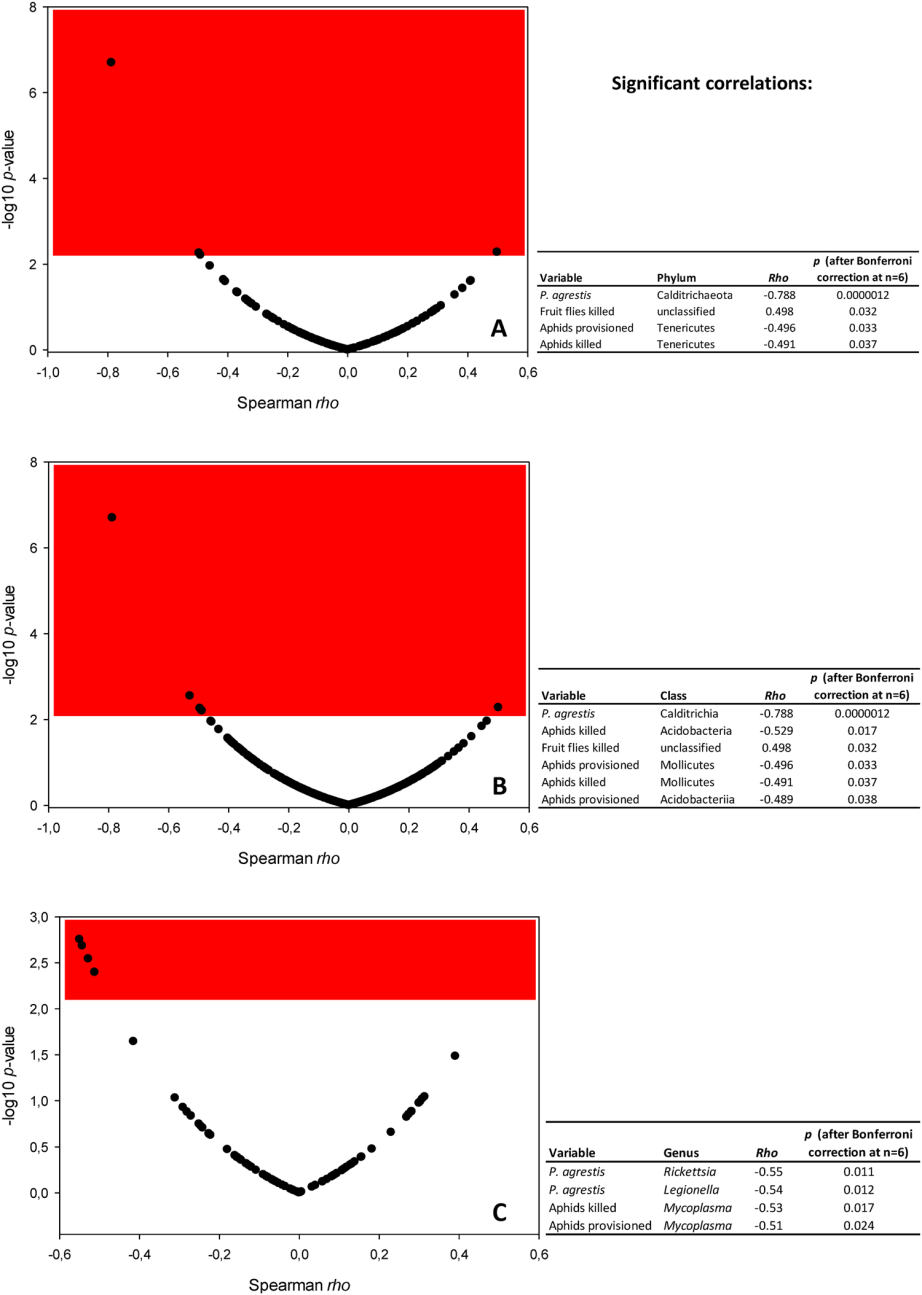
Volcano plots for Spearman correlations between the environmental variables (provisioned food, frequency of any provisioned food, *P. agrestis* / *P. palustris*, number of provisioned aphids, number of provisioned fruit flies, number of killed aphids, number of killed fruit flies, traveled distance) and bacterial and archaeal phyla (**A**), classes (**B**), or selected genera of obligate or facultative endosymbionts (**C**). The lists of tested bacterial and archaeal classes, bacterial and archaeal phyla, and genera of endosymbionts are provided in Tables S2, S1, and S3, respectively. The red area indicates significant correlations (− log *p* < 0.05 after applying Bonferroni correction at n = 6). Significant variables (*p* < 0.05 after applying Bonferroni correction at n = 6) are shown in tables accompanying each plot.

**Figure 3:**
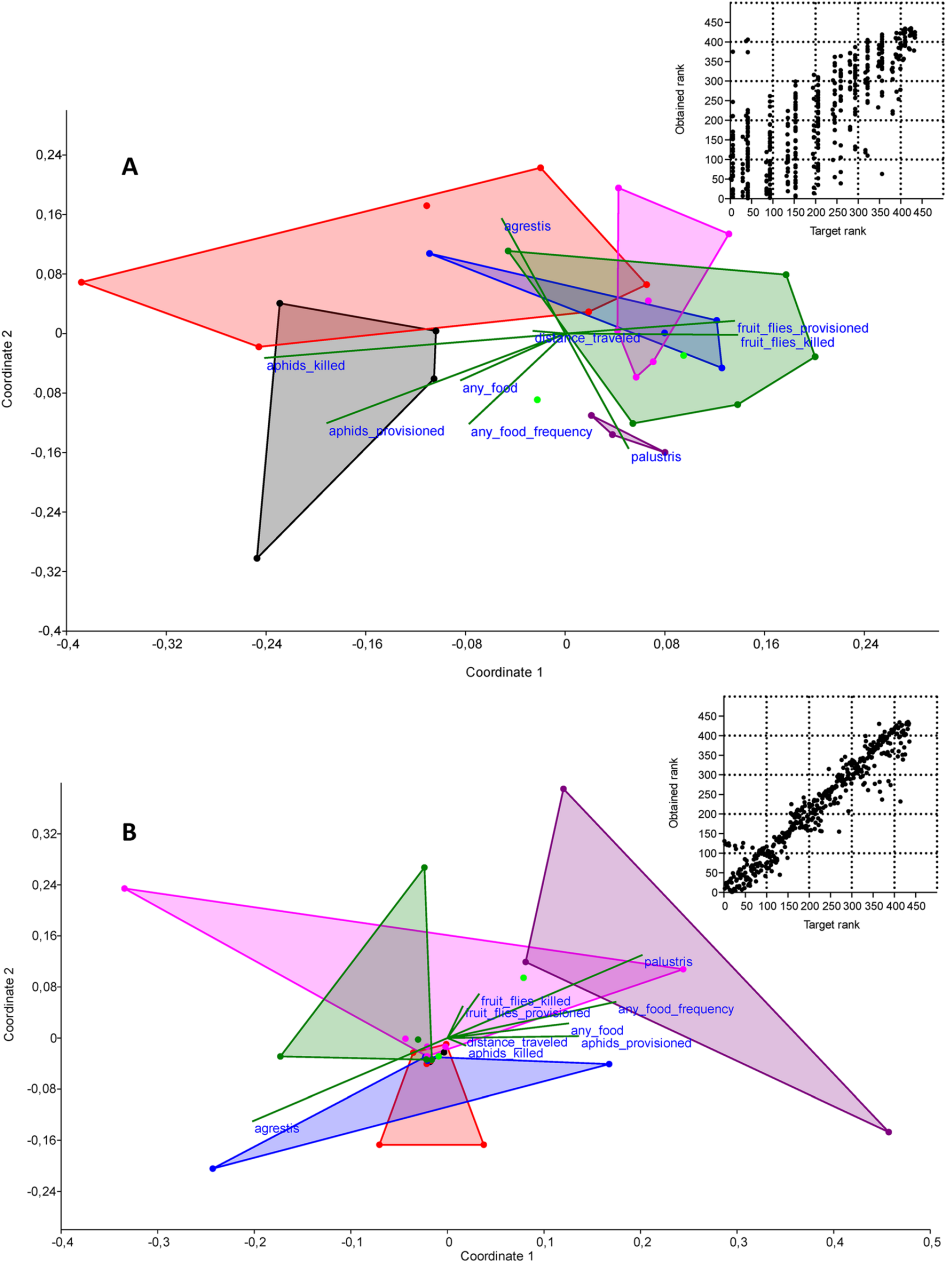
NMDS plots of the effects of environmental variables (provisioned food, frequency of any provisioned food, *P. agrestis*/*P. palustris*, number of provisioned aphids, number of provisioned fruit flies, number of killed aphids, number of killed fruit flies, traveled distance) on the analyzed microbial endosymbionts. (**A**) NMDS (Kimura similarity measure) of bacterial and archaeal phyla; stress ratio 0.3363; R^2^ (axis 1) 0.000; R^2^ (axis 2) 0.0117. (**B**) NMDS (Gower similarity measure) of the relative abundance of selected genera of obligate or facultative endosymbionts; stress ratio 0.1181; R^2^ (axis 1) 0.7152; R^2^ (axis 2) 0.6924. The Shepard plots (inserts) suggested low concordance of obtained and target ranks in (**A**) but high concordance in (**B**). Grey = *P. agrestis*, provisioned with aphids twice daily; red = *P. agrestis*, provisioned with aphids once daily; blue = *P. agrestis*, starved control; pink = *P. agrestis*, provisioned with fruit flies twice daily; dark green = *P. agrestis*, provisioned with fruit flies once daily; violet = *P. palustris*, provisioned with aphids twice daily; light green = *P. palustris*, provisioned with fruit flies once daily. Points show host individuals; convex hulls indicate host individuals of the same type.

In both spider species, the most abundantly detected was Proteobacteria (65.9% and 63.2% of reads in *P. agrestis* and *P. palustris*, respectively), followed by Firmicutes (14.2% and 16.3%) and Actinobacteria (9.7% and 8.3%) (Table S1). Relatively rare were Bacteriodetes (1.3% and 1.6%), Spirochaetes (0.04% and 0.08%), Verrumicrobia (0.05% and 0.01%), and Euryarchaeota (0.01% and none). There were negligible differences between the spiders treated with the tested food regimens (one-way PERMANOVA (Euclidean distance measure): permutation N = 9999, total sum of squares 1.2 × 10^10^, within-group of squares 8.8 × 10^9^, F = 1.259, *p* > 0.05). Subsequent pairwise comparisons revealed that the only significant differences were detectable when comparing *P. agrestis* fed fruit flies twice daily with the control (F = 8.8, *p* = 0.02) or with *P. agrestis* fed fruit flies once daily (F = 5.0, *p* = 0.047). At the level of individual phyla, the only prominent correlations consisted of the lower relative abundance of Calditrichaeota in *P. agrestis* than in *P. palustris* (*rho* = − 0.79, *p*_(Bonf. corr. n=6)_ < 0.001), and the lower relative abundance of Tenericutes in spiders provided with more aphids or in those that killed more aphids (*rho* = − 0.50, *p*_(Bonf. corr. n=6)_ = 0.03, and *rho* = − 0.49, *p*_(Bonf. corr. n=6)_ = 0.04, respectively) (Fig. 2A). The results of the NMDS are shown in Fig. 3A. The number of phyla reached 13.5 ± 0.4 (range 8–18) phyla per host individual. The diversity of phyla strongly differed when comparing *P. agrestis* fed fruit flies once and twice daily—the Gini-Simpson index was higher in spiders fed once daily (0.7 ± 0.1 vs. 0.4 ± 0.1), whereas the Simpson index, Shannon H, Brillouin, and Fisher’s alpha were higher in spiders fed twice daily (one-tailed *t*-test p < 0.05 each). In contrast, there were no such differences found between *P. agrestis* fed aphids once or twice daily (one-tailed *t*-test *p* > 0.05 each).

At the level of individual classes, Calditrichia were less abundant in *P. agrestis* than in *P. palustris* (*rho* = -0.79, *p*_(Bonf. corr. n=6)_ < 0.001). The relative abundance of Acidobacteria and Mollicutes was inversely related to the number of killed aphids (*rho* = − 0.53, *p*_(Bonf. corr. n=6)_ = 0.02, and *rho* = − 0.49, *p*_(Bonf. corr. n=6)_ = 0.04, respectively). Both these classes were also less abundant in spiders that were provided with more aphids (*rho* = − 0.49, *p*_(Bonf. corr. n=6)_ = 0.04, and *rho* = − 0.50, *p*_(Bonf. corr. n=6)_ = 0.03, respectively) (Fig. 2B). The number of classes reached 26.5 ± 0.9 (range 12–35) classes per host individual. The diversity of classes differed strongly when comparing *P. agrestis* fed fruit flies once and twice daily—the Gini-Simpson index was higher in spiders fed once daily (0.7 ± 0.1 vs. 0.3 ± 0.0), whereas the Simpson index, Shannon H, Brillouin, and Fisher’s alpha were higher in spiders fed twice daily (one-tailed *t*-test p < 0.05 each). In contrast, there were no such differences found between *P. agrestis* fed aphids once or twice daily (one-tailed *t*-test *p* > 0.05 each).

### Description of facultative and obligate endosymbionts identified in each predator species

We found 324 OTUs that are obligately or facultatively endosymbiotic. Vertically transmitted endosymbionts, which are frequently associated with the reproductive manipulation of their arthropod hosts, were rare. Two OTUs were assigned to *Wolbachia pipientis* (they were present in eight *P. agrestis* individuals and one *P. palustris* individual), and one OTU represented *Rickettsia* sp. H820 (in two *P. agrestis* individuals and three *P. palustris* individuals). The genus *Cardinium* was absent from the analyzed datasets.

Additional strains of endosymbiotic OTUs were represented by 31 OTUs of *Rhabdochlamydia*, 177 OTUs of *Rickettsiella*, 52 OTUs of facultative intracellular parasite *Legionella*, seven OTUs of *Spiroplasma*, 32 OTUs of *Mycoplasma*, and several other endosymbiotic Chlamydiia and Alphaproteobacteria. The obligate or facultative endosymbionts were present in all analyzed spider individuals. All tested positive for 4.9 ± 0.4 genera of obligate or facultative endosymbionts (range one to nine genera). The number of OTUs of obligate or facultative endosymbionts was 16.8 ± 5.5 OTUs (range 2–174 OTUs) of obligate or facultative endosymbionts per host individual.

### Link between provisioned prey and diversity of endosymbionts

There were negligible differences in obligate or facultative endosymbionts between the spiders treated with the tested food regimens (one-way PERMANOVA (Euclidean distance measure): permutation N = 9999, total sum of squares 3.9 × 10^9^, within-group of squares 3.4 × 10^9^, F = 0.598, *p* > 0.05). Subsequent pairwise comparisons revealed that the only significant differences were detectable when comparing *P. agrestis* and *P. palustris*, both fed aphids twice daily (F = 10.0, *p* = 0.03). At the level of individual genera, the only significant correlations were the lower relative abundance of *Legionella* spp. and *Rickettsia* spp. in *P. agrestis* when compared to *P. palustris* (*rho* = − 0.54, *p*_(Bonf. corr. n=6)_ = 0.01, and *rho* = − 0.55, *p*_(Bonf. corr. n=6)_ = 0.01, respectively), and the relative abundance of *Mycoplasma* spp. was negatively correlated with the provisioned or killed aphids (*rho* = − 0.51, *p*_(Bonf. corr. n=6)_ = 0.02, and *rho* = − 0.53, *p*_(Bonf. corr. n=6)_ = 0.02, respectively) (Fig. 2C). The results of the NMDS are shown in Fig. 3B.

## Discussion

We found that *P. agrestis* consumed a higher total amount of prey when provisioned twice daily instead of once daily. This observation applied both to the number of killed aphids as well as fruit flies. Generally, the number of killed prey was high, and numerous previous studies suggested that this overkilling behavior (killing the prey without subsequent feeding or discarding partially consumed prey) serves as an adaptation to food-limited environments^57–60^. Massive overkilling of up to 65% of the prey is also known for *Pardosa* spiders^59,61^. Therefore, the high number of killed prey when provisioned once or twice daily may correspond to the artificially induced food limitation of the starved spiders.

Starved (control) spiders displayed lower locomotor activity than those provided with the prey. The measurements were not performed at a time when prey was present; therefore, they cannot be classified as prey-hunting behavior. The wolf spiders are sit-and-wait^62^ or sit-and-move^63^ predators. Therefore, they hunt by sitting still but frequently change their hunting position. Locomotor activity of *Pardosa* spp. increases with temperature^64^ and low humidity^65^ and may decline after exposure to neurotoxic agrochemicals, such as neonicotinoids^66^, with age^67^, or when exposed to cues from relatives^68^. Interestingly, in another main group of arthropod predators, carabid beetles, hunger has been proposed to keep individuals active even outside their main activity periods^69^. In contrast, a study on the wolf spider *Schizocosa ocreata* did not identify any effects of hunger on patch residence time^70^. Importantly, starved wolf spiders *Lycosa lenta* decreased their metabolic rates by 30–40%^71^, which may partially explain the observed decrease in locomotor activity.

The tissue-resident microbiomes of both analyzed spider species were diverse, dominated by Proteobacteria (> 60% reads), Firmicutes, and Actinobacteria. Relatively rare were Bacteroidetes, Spirochaetes, Verrumicrobia, and Euryarchaeota. These results differ from those obtained previously in other spider species. For example, *A. bruennichi* tissues were dominated by a single unknown symbiont proposed to represent a novel clade (over 90% of reads in legs or prosoma)^34^. Cephalothorax of five Theridiidae spp. did not share any dominant phyla or genera across the five tested species. *Latrodectus geometricus* was dominated by *Candidatus* Rhabdochlamydia, while *Gilliamella* was a major bacterial symbiont that was found across the other spider species, particularly *Latrodectus mactans* and *Latrodectus hesperus*^35^. Comparative data from spiders are rare, and additional tissue-specific analyses beyond the examination of gut content and whole spider bodies are needed.

Regarding inherited endosymbionts, only some of the analyzed spider individuals were infected with *Wolbachia* and *Rickettsia*, in line with previous reports from other arthropod taxa. More than half of arthropod species are estimated to be infected with *Wolbachia*, approximately a quarter are infected with *Rickettsia*, and one-eighth of species are infected with *Cardinium*^72^. However, the frequency of infection within a given species is often low. The majority of *Wolbachia*-infected arthropod species (n_total_ = 488) have less than half of their individuals infected. This observation also applies to *Rickettsia* (81% of insect species known to be infected, n_total_ = 54) and *Cardinium* (87% of insect species known to be infected, n_total_ = 31)^73^. In some orders, less than 10% of individuals were infected. These orders include Coleoptera, in which the infection rate by *Wolbachia* is below 5%. A similar pattern applies to Hemiptera, Diptera, Odonata, and Ephemeroptera^73^. The data from spiders are scarce—the study by Dunaj et al.^35^ examined only three to four individuals per species and concluded that *Wolbachia* was present in all examined individuals of three *Latrodectus* spp. and *Steatoda grossa* but not *Parasteatoda tepidariorum*. In *Oedothorax gibbosus*, 42–44% of females and 57–64% of males were found to be infected by *Wolbachia* in two regions of Belgium^40^. The most extensive study in this regard was conducted by Duron et al.^38^, who found *Wolbachia* to be absent in many spider families, including Amaurobiidae (one species), Araneidae (seven species), Dysderidae (one species) and Pisauridae (one species). They also tested four species of Lycosidae. Two of these, *Alopecosa pulverulenta* and *Pardosa pullata*, were *Wolbachia*-positive (25% and 5% of individuals), whereas *Pardosa lugubris* and *Pardosa purbeckensis* were *Wolbachia*-negative. In other *Wolbachia*-positive spider species, the infection rates by *Wolbachia* ranged from 5% (*Pachygnatha degenerii* and *Enoplognatha ovata*) to 95% (*Pholcus phalangioides*), but most were below 50%^38^.

The increased frequency of feeding with fruit flies, but not aphids, increased the dominance and decreased the alpha diversity of OTUs. However, there were no major changes at the level of individual phyla, classes, or genera. The literature on this topic is scarce, as most of the feeding-related papers that focused on spider microbiomes focused on the gut microbiome or analyzed the microbiome of the whole abdomen (including the gut contents). Environmental factors, such as water precipitation, were found to drive the relative abundance of *Mycoplasma* and *Proteus* in *Stegodyphus dumicola*^46^. Exposure to increased loads of the cuticular bacteria *Bacillus thuringiensis*, *Microbacterium oxydans*, and *Pantoea* sp. led to quicker prey-attacking reactions and facilitated cooperativity in the same spider species^47^. A manipulative experimental study is needed to answer the question of whether the food composition and food quantity have any effects on the species richness and relative abundance of endosymbionts and whether there exists a feedback loop that leads to higher predation rates or higher locomotor activity. These tests should focus particularly on changes in dietary intake of proteins and lipids or the prey quantity itself and further on additive or multiplicative effects of mitochondria-targeting insecticides and perhaps some other agrochemicals.

In conclusion, we uncovered a highly complex tissue-resident microbiome of two agrobiont spider species. The composition of the microbiome was unique, and the shares of individual phyla differed strongly from those reported from the guts of various spider species and from those obtained by sequencing whole spiders. Furthermore, we provided conclusive evidence of the presence of inherited endosymbionts and on their infection frequencies. We found that the composition of the tissue-resident microbiome is robust, is not sensitive to short-term changes in feeding, and does not project into the number of killed prey and locomotor activity.

## Supplementary Information

Supplementary Information.

## Data Availability

All data generated or analyzed during this study are included in this published article and its supplementary information files. Raw 16S rDNA reads were deposited in the NCBI Sequence Read Archive (SRA) database (BioProject ID PRJNA910212; accession file SUB12381240; direct link: http://www.ncbi.nlm.nih.gov/bioproject/910212).
