# Supplementary material for: Food provisioning to Pardosa spiders decreases the levels of tissue-resident endosymbiotic bacteria
Source: Sci Rep. 2023 Apr 28;13:6943. doi: 10.1038/s41598-023-34229-1 (PMC10147729; doi:10.1038/s41598-023-34229-1)

SUPPLEMENTARY MATERIALS

Food provisioning to *Pardosa* spiders decreases the levels of tissue-resident endosymbiotic bacteria

Milan Řezáč, Veronika Řezáčová, Nela Gloríková, Ema Neméthová, Petr Heneberg

Table S1. Relative share [%] of reads of archaeal and bacterial phyla in analyzed *P. agrestis* and *P. palustris* cephalothorax and legs. The color code is used to indicate the relative abundance, with the highest abundance shown in red and the lowest abundance shown in blue.

Kingdom	Phylum	*P. agrestis*	*P. palustris*
Archaea	Euryarchaeota	0.01	0.00
Archaea	Thaumarchaeota	0.01	0.01
Bacteria	Acidobacteria	0.10	0.07
Bacteria	Actinobacteria	9.73	8.33
Bacteria	Armatimonadetes	0.00	0.00
Bacteria	Bacteroidetes	1.29	1.61
Bacteria	Calditrichaeota	0.00	0.05
Bacteria	Candidatus Melainabacteria	0.01	0.02
Bacteria	Cyanobacteria	0.39	0.86
Bacteria	Deinococcus-Thermus	0.04	0.00
Bacteria	Firmicutes	14.22	16.29
Bacteria	Fusobacteria	0.02	0.04
Bacteria	Gemmatimonadetes	0.06	0.11
Bacteria	Chlamydiae	2.02	0.16
Bacteria	Chloroflexi	0.03	0.03
Bacteria	Lentisphaerae	0.02	0.00
Bacteria	Planctomycetes	0.15	0.08
Bacteria	Proteobacteria	65.87	63.20
Bacteria	Rhodothermaeota	0.10	0.13
Bacteria	Spirochaetes	0.04	0.08
Bacteria	Synergistetes	0.00	0.00
Bacteria	Tenericutes	0.22	0.26
Bacteria	Thermotogae	0.00	0.00
Bacteria	unclassified	5.61	8.67
Bacteria	Verrucomicrobia	0.05	0.01

Table S2. Relative share [%] of reads of archaeal and bacterial classes in analyzed *P. agrestis* and *P. palustris* cephalothorax and legs. The color code is used to indicate the relative abundance, with the highest abundance shown in red and the lowest abundance shown in blue.

Kingdom	Phylum	Class	*P. agrestis*	*P. palustris*
Archaea	Euryarchaeota	Halobacteria	0.0028	0.0000
Archaea	Euryarchaeota	Methanobacteria	0.0057	0.0000
Archaea	Thaumarchaeota	Nitrososphaeria	0.0076	0.0073
Bacteria	Acidobacteria	Acidobacteriia	0.0797	0.0728
Bacteria	Acidobacteria	Blastocatellia	0.0193	0.0009
Bacteria	Actinobacteria	Acidimicrobiia	0.0386	0.0278
Bacteria	Actinobacteria	Actinomycetia	9.3694	8.1168
Bacteria	Actinobacteria	Coriobacteriia	0.0167	0.0000
Bacteria	Actinobacteria	Rubrobacteria	0.1688	0.0981
Bacteria	Actinobacteria	Thermoleophilia	0.1342	0.0891
Bacteria	Armatimonadetes	Fimbriimonadia	0.0016	0.0000
Bacteria	Bacteroidetes	Bacteroidia	0.2225	0.2313
Bacteria	Bacteroidetes	Cytophagia	0.2541	0.5946
Bacteria	Bacteroidetes	Flavobacteriia	0.4440	0.5586
Bacteria	Bacteroidetes	Chitinophagia	0.1540	0.0317
Bacteria	Bacteroidetes	Saprospiria	0.0131	0.0608
Bacteria	Bacteroidetes	Sphingobacteriia	0.2007	0.1285
Bacteria	Calditrichaeota	Calditrichia	0.0002	0.0476
Bacteria	*Candidatus* Melainabacteria	undefined *Candidatus* Melainabacteria	0.0050	0.0167
Bacteria	Cyanobacteria	Cyanobacteria	0.3853	0.7715
Bacteria	Cyanobacteria	unclassified	0.0002	0.0917
Bacteria	*Deinococcus-Thermus*	Deinococci	0.0367	0.0009
Bacteria	Firmicutes	Bacilli	12.2729	14.8730
Bacteria	Firmicutes	Clostridia	1.3401	0.9176
Bacteria	Firmicutes	Erysipelotrichia	0.0479	0.0951
Bacteria	Firmicutes	Negativicutes	0.0948	0.0000
Bacteria	Firmicutes	Tissierellia	0.4529	0.3980
Bacteria	Firmicutes	unclassified	0.0002	0.0000
Bacteria	Firmicutes	undefined Firmicutes	0.0188	0.0026
Bacteria	Fusobacteria	Fusobacteriia	0.0211	0.0364
Bacteria	Gemmatimonadetes	Gemmatimonadetes	0.0623	0.1135
Bacteria	Chlamydiae	Chlamydiia	2.0237	0.1615
Bacteria	Chloroflexi	Anaerolineae	0.0107	0.0283
Bacteria	Chloroflexi	Caldilineae	0.0039	0.0021
Bacteria	Chloroflexi	Dehalococcoidia	0.0015	0.0000
Bacteria	Chloroflexi	Ktedonobacteria	0.0005	0.0000
Bacteria	Chloroflexi	Thermomicrobia	0.0136	0.0000
Bacteria	Lentisphaerae	Lentisphaeria	0.0198	0.0017
Bacteria	Lentisphaerae	Oligosphaeria	0.0008	0.0000
Bacteria	Planctomycetes	Phycisphaerae	0.0693	0.0000
Bacteria	Planctomycetes	Planctomycetia	0.0857	0.0848
Bacteria	Proteobacteria	Acidithiobacillia	0.0024	0.0004
Bacteria	Proteobacteria	Alphaproteobacteria	6.5573	2.8814
Bacteria	Proteobacteria	Betaproteobacteria	2.7385	2.0087
Bacteria	Proteobacteria	Deltaproteobacteria	0.2077	0.3196
Bacteria	Proteobacteria	Epsilonproteobacteria	0.0135	0.0000
Bacteria	Proteobacteria	Gammaproteobacteria	56.2056	57.9847
Bacteria	Proteobacteria	Oligoflexia	0.1436	0.0021
Bacteria	Rhodothermaeota	Rhodothermia	0.1014	0.1251
Bacteria	Spirochaetes	Spirochaetia	0.0404	0.0814
Bacteria	Synergistetes	Synergistia	0.0023	0.0004
Bacteria	Tenericutes	Mollicutes	0.2204	0.2605
Bacteria	Thermotogae	Thermotogae	0.0005	0.0000
Bacteria	unclassified	unclassified	5.6141	8.6686

Bacteria	Verrucomicrobia	Opitutae	0.0120	0.0047
Bacteria	Verrucomicrobia	Terrimicrobia	0.0320	0.0000
Bacteria	Verrucomicrobia	Verrucomicrobiae	0.0076	0.0009

Table S3. Relative share [%] of reads of selected genera of obligate or facultative endosymbionts in analyzed *P. agrestis* and *P. palustris* cephalothorax and legs. The color code is used to indicate the relative abundance, with the highest abundance shown in red and the lowest abundance shown in blue.

**Class**	***P. agrestis***	***P. palustris***
Wolbachia	0.8828	0.0650
unclassified *Chlamydiia*	0.2493	4.5145
Spiroplasma	0.3721	5.1640
Rickettsiella	80.8259	5.8785
Rickettsia	0.0024	4.6768
Rhabdochlamydia	14.4535	3.7675
Parachlamydia	0.0340	0.0000
Neochlamydia	0.1520	3.2153
Mycoplasma	0.7746	10.8152
Legionella	2.1390	61.2212
Candidatus Metachlamydia	0.0207	0.0000
Candidatus Mesochlamydia	0.0924	0.5846
Candidatus Cyrtobacter	0.0000	0.0974
Anaplasma	0.0012	0.0000

Fig. S1. Rarefaction of datasets of Bacteria and Archaea obtained from *P. agrestis* and *P. palustris*. Sample rarefaction of OTUs from *P. agrestis* (A) and *P. palustris* (B), and individual rarefaction of phyla from these two host species (C).

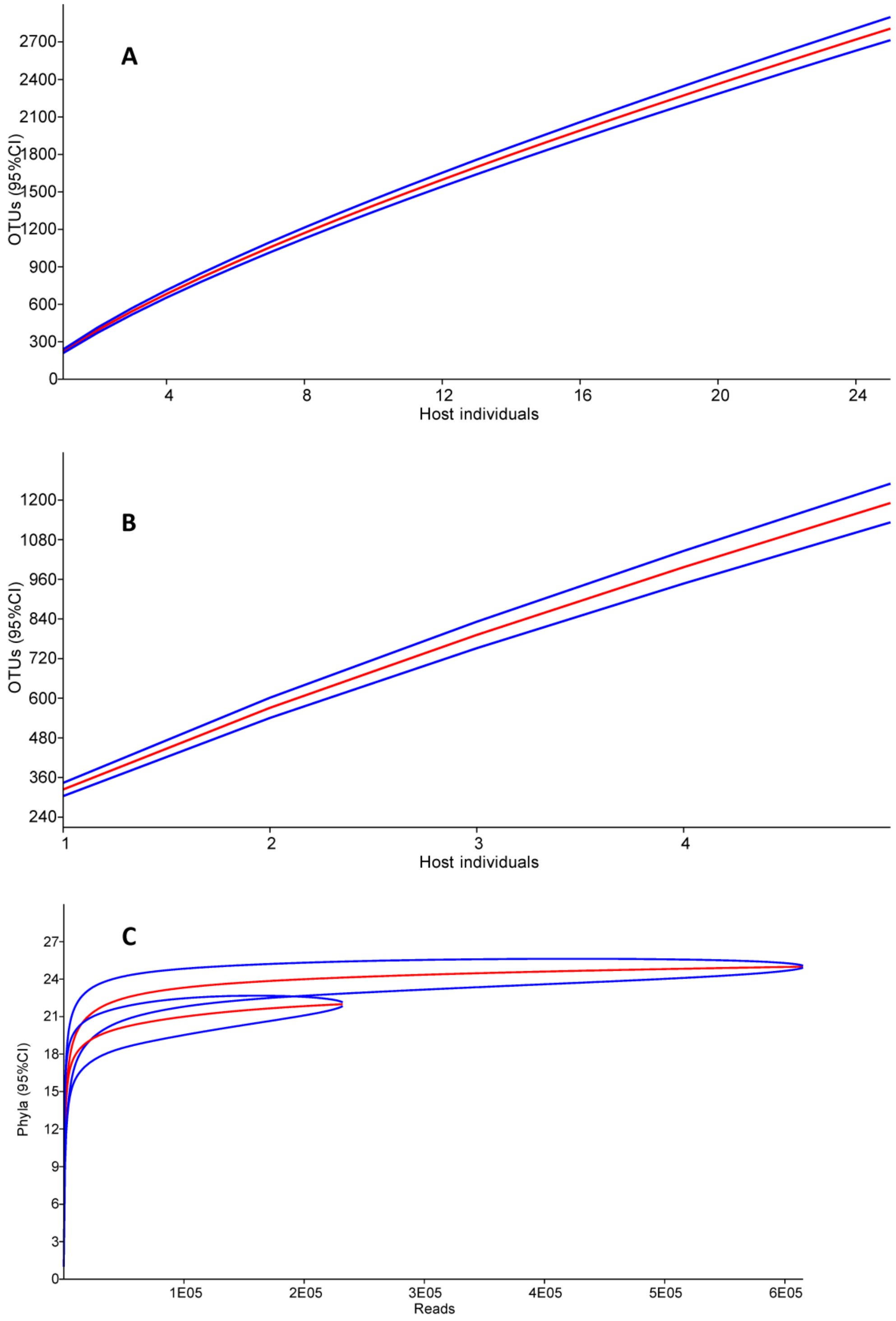

Supplement: Supplementary file 1 — Supplementary Information. [file 41598_2023_34229_MOESM1_ESM.pdf]
